# IMPLEMENTATION OF MULTIMODAL INTERVENTIONS FOR CAREGIVERS OF OLDER ADULTS WITH VISUAL IMPAIRMENTS

**DOI:** 10.1093/geroni/igac059.534

**Published:** 2022-12-20

**Authors:** Afeez Hazzan, Pamela Haibach-Beach, Lauren Lieberman

**Affiliations:** State University of New York at Brockport, Brockport, New York, United States; State University of New York at Brockport, Brockport, New York, United States; State University of New York at Brockport, Brockport, New York, United States

## Abstract

There is a paucity of research on the impact of caregiving on the quality of life of unpaid caregivers for older adults aged 60 years or older with visual impairments (VI). The purpose of this study is to test multimodal interventions to improve quality of life and well-being in unpaid caregivers of older adults with VI. The objectives were: (1) to implement multimodal interventions targeted towards improving the quality of life of family caregivers of older adults with a VI; (2) to evaluate the efficacy/effectiveness of the interventions in improving the quality of life of older adults with a VI. The outcomes of interest include: quality of life, health, stress, burden, problem-solving, and barriers. For this study, a 10-week virtual intervention was implemented with 12 caregivers and eight older adults with visual impairments, for a total of 20 participants. The intervention was held for one hour for 10 consecutive weeks and included activities such as tai chi, yoga, meditation, music, nutrition, and other de-stressing techniques. Participants completed a series of questionnaires before and after the intervention period. These questionnaires include: Satisfaction with Life Scale, Living Arrangement and Indicators of Social Interaction survey, Caregiver quality of life (EQ-5D), the Perceived Change Index, and the Geriatric Depression Scale. Findings from the data analysis show excellent caregiver engagement throughout, including participation in the pre and post surveys. Results show the benefits of multimodal interventions for caregivers as well as older adults with VI. Future studies should focus on interventions that are most promising.

